# Biomaterial-driven in situ cardiovascular tissue engineering—a multi-disciplinary perspective

**DOI:** 10.1038/s41536-017-0023-2

**Published:** 2017-06-16

**Authors:** Tamar B. Wissing, Valentina Bonito, Carlijn V. C. Bouten, Anthal I. P. M. Smits

**Affiliations:** 1 Department of Biomedical Engineering, Eindhoven University of Technology, Eindhoven, The Netherlands; 2 Institute for Complex Molecular Systems, Eindhoven University of Technology, Eindhoven, The Netherlands

## Abstract

There is a persistent and growing clinical need for readily-available substitutes for heart valves and small-diameter blood vessels. In situ tissue engineering is emerging as a disruptive new technology, providing ready-to-use biodegradable, cell-free constructs which are designed to induce regeneration upon implantation, directly in the functional site. The induced regenerative process hinges around the host response to the implanted biomaterial and the interplay between immune cells, stem/progenitor cell and tissue cells in the microenvironment provided by the scaffold in the hemodynamic environment. Recapitulating the complex tissue microstructure and function of cardiovascular tissues is a highly challenging target. Therein the scaffold plays an instructive role, providing the microenvironment that attracts and harbors host cells, modulating the inflammatory response, and acting as a temporal roadmap for new tissue to be formed. Moreover, the biomechanical loads imposed by the hemodynamic environment play a pivotal role. Here, we provide a multidisciplinary view on in situ cardiovascular tissue engineering using synthetic scaffolds; starting from the state-of-the art, the principles of the biomaterial-driven host response and wound healing and the cellular players involved, toward the impact of the biomechanical, physical, and biochemical microenvironmental cues that are given by the scaffold design. To conclude, we pinpoint and further address the main current challenges for in situ cardiovascular regeneration, namely the achievement of tissue homeostasis, the development of predictive models for long-term performances of the implanted grafts, and the necessity for stratification for successful clinical translation.

## Introduction

Cardiovascular tissues, such as heart valves and blood vessels, are sophisticated dynamic tissues that can grow and adapt their structure according to the hemodynamic environment in which they function. It is this characteristic quality that makes it notoriously challenging to replace these tissues with artificial substitutes in case of end-stage disease or damage. For small-diameter arteries (e.g., peripheral arteries, arteriovenous shunts) and the semilunar heart valves in particular, the development of living, adaptive replacement tissues could greatly improve the underachieving current artificial replacements. Novel cardiovascular tissue engineering (TE) strategies are increasingly moving from an in vitro to an in situ approach. In situ TE is defined as biomaterial-induced endogenous regeneration directly in the tissue’s functional site, or in situ, starting from readily-available, resorbable grafts that gradually transform into an autologous, homeostatic replacement tissue with the ability to repair, remodel, and grow. Grafts for in situ TE can be of biological or synthetic nature and either acellular or on-the-fly preseeded. However, prerequisite for this approach is that the graft is readily-available for implantation and that the graft allows for colonization and remodeling by host cells in order to achieve an adaptive autologous tissue over time. Whereas the traditional TE dogma comprises labor-intensive and lengthy in vitro culture and conditioning phases, in situ TE has been proposed as a more cost-effective and on-demand approach, using relatively simple and shelf-ready grafts.^1–3^

**Fig. 1:**
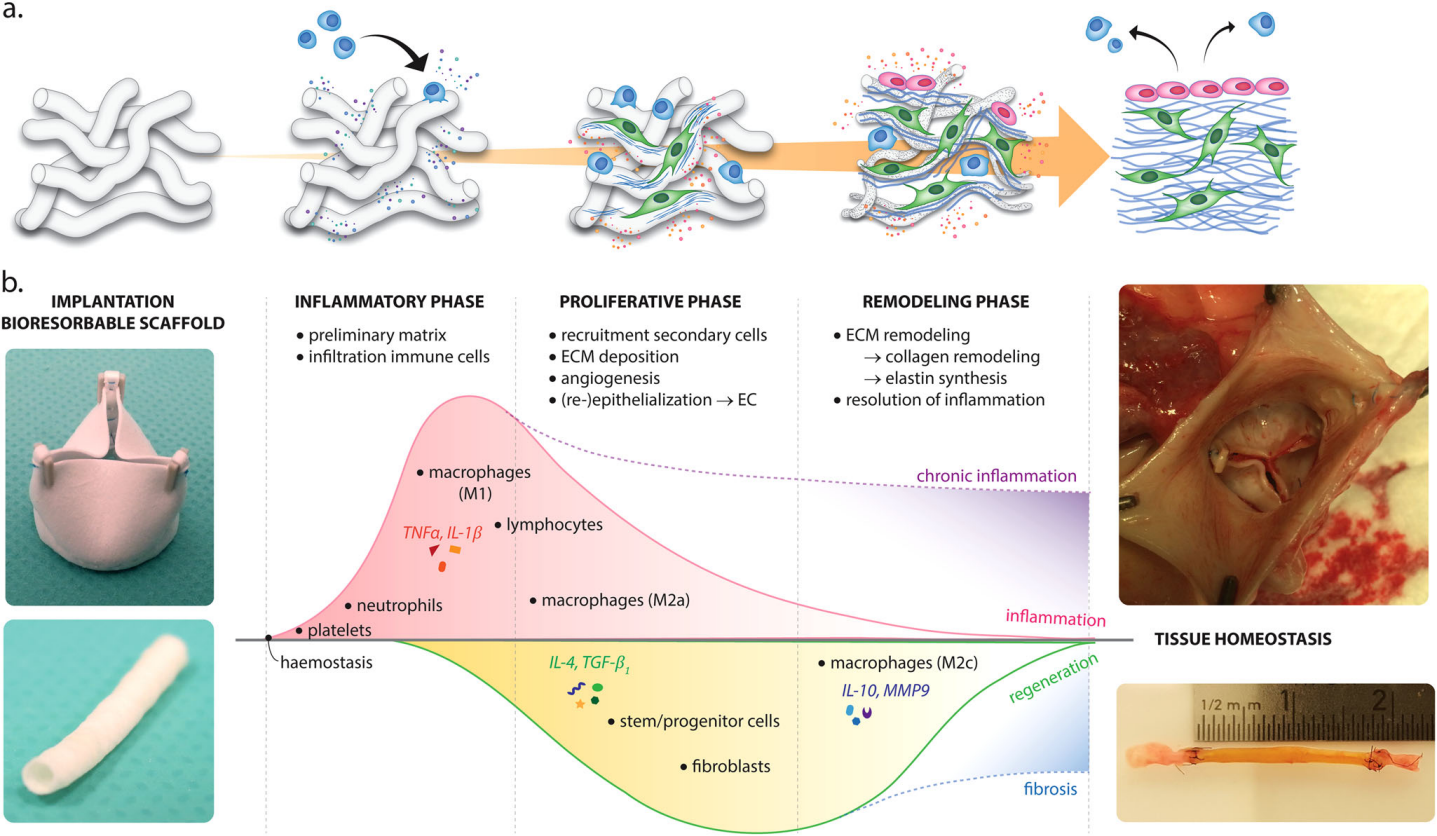
Overview of the different stages of in situ tissue regeneration, going from a synthetic, biodegradable bare construct toward a viable substitute (**a**). Although many aspects underlying in situ regeneration remain unknown, it is hypothesized that the stages mirror the natural phases of the wound healing response (**b**), starting with the inflammatory phase, characterized by the infiltration of immune cells and the formation of a preliminary matrix. The subsequent proliferative phase is characterized by a secondary influx of immune and tissue producing cells, extracellular matrix (ECM) deposition, angiogenesis and (re-)endothelialization of the construct. Tissue homeostasis is restored after a remodeling phase of the newly formed ECM and the resolution of inflammation. Photographs of heart valves adapted from;^33^ photographs of vascular grafts courtesy of Renée Duijvelshoff

The approach of in situ TE is built on the notion that the natural inflammatory response can be harnessed to induce endogenous tissue regeneration (Fig. 1). The resorbable immunomodulatory scaffold provides a temporary microenvironment, which functions as an instructive road map for endogenous cells to infiltrate and create new, living, and functional tissue. It is hypothesized that, upon implantation, the scaffold provides support for mature tissue formation and adequate mechanical properties to withstand the hemodynamic loads. Over time, the scaffold should slowly resorb, ultimately resulting in a purely biological structure which has the ability to repair, remodel, and grow. Proof-of-concept has been demonstrated by milestone studies describing the endogenous regeneration of small-diameter blood vessels and heart valves using acellular synthetic scaffolds or de novo engineered decellularized extracellular matrix (ECM) (see Table 1). Despite these encouraging reports, we are only just beginning to grasp a more fundamental understanding of the biomaterial-driven regeneration in the complex hemodynamic environment, and many key questions remain to be answered:

- How can a scaffold recapitulate the complex layered architecture of cardiovascular tissues?
- What is the influence of biomechanical stimuli on the organization of new tissue?
- Is it necessary to incorporate exogenous bioactivities to enhance the robustness of the in situ TE approach?
- How fast and by which mechanism should a scaffold degrade?
- Is this patient-dependent and should this be personalized?
- How can we predict inflammatory-driven functional regeneration?

**Table 1:** Selection of recent in vivo studies applying in situ tissue engineered vascular grafts

Primary research target	Material	Design	Model	Main findings	Ref.
Mechanisms of in situ regeneration	PGA + P(CL-LA)	Large pore (45 µm) non-woven mesh + BMC in fibrin	Mouse (IVC)	In situ regeneration is an inflammation-driven process. Preseeded BMC have paracrine function via MCP-1	19
	PGA or PLA + P(CL-LA)	Large pore non-woven mesh ± BMC in fibrin	Mouse (IVC or IA)	Temporal characterization of ECM formation, remodeling and resulting biomechanical properties in venous and arterial circulation	159–161, 189
	PCL + fibrin ± MCP-1	Electrospun grafts shielded with GoreTex sheaths	Rat (AA)	Circulatory cells have regenerative capacity, which can be boosted by MCP-1	36
Degradation	PCL/Chitosan	Electrospun nanofibrous grafts	Mouse (IA), Sheep (CA)	Fast degradation of chitosan as “sacrifical material” helps regeneration	190
	PLA-PGA or PLA + P(CL-LA)	Microfibrous graft + nanofibrous outer layer	Mouse (IA)	Fast degradation prevents calcification in contrast to slow degrading grafts	162
	PCL	Micro/nano-fibrous grafts	Rat (AA)	Insufficient regeneration and calcification at long-term (18 months) follow-up	131
	PGS/heparin + PCL sheath	Porous PGS/heparin core with dense non-woven PCL sheath	Rat (AA)	Timely degradation and mechanotransduction important for regeneration	22
Micro-structure	PLA-PLCL or PLA	Large-pore (30 µm) vs. small-pore (5 µm) grafts	Mouse (IA)	Large-pore grafts prevent calcification in comparison to small-pore grafts	191
	PLCL + PLA	Large-pore (29 µm) vs. small-pore (13 µm) PLCL core with PLA reinforcement	Mouse (IA)	No effect of pore size in terms of cell infiltration, macrophage polarization and tissue formation. No aneurysms	192
	PCL/PEO	Three-layered graft with a large-pore (26 µm) inner layer with dense outer and thin dense inner layers	Rabbit (CA)	Porous middle layer leads to better cell infiltration, faster degradation and improved regeneration, proposedly due to increased MCP-1 and VEGF	193
	PCL	Large-pore (30 µm) microfbrous vs. small-pore (2 µm) nanofibrous grafts	Rat (AA)	Large pores promote M2 macrophage polarization, enhanced cell infiltration and regeneration	194
	PCL	Electrospun nano-/microfibrous bilayered grafts	Rat (AA)	Layered grafts are a tool to control cell infiltration and regeneration	116
	PCL	Inner layer of circumferentially aligned microfibers + random nanofibrous outer layer	Rat (AA)	Contact guidance by aligned fibers stimulates native-like SMC organization	195
Mechanical properties	TPU	Electrospun, microfibrous graft	Rat (AA)	Thin-walled graft with adequate biomechanical properties	196
	PCL/elastin	Multi-layered electrospun graft	Rabbit (CA)	Native-like mechanical properties using recombinant elastin	104
	PGS + PCL sheath	Porous PGS reinforced by PCL sheath with varying densities	Rat (AA)	Variation of the outer sheath density directly influences regeneration (SMC organization and M2 polarization)	197
	PGA + P(CL-LA)	Non-woven porous mesh	Mouse (IVC) + in silico model	Biomechanical diversity between 12 and 24 months of implantation due to variations in the ratio collagen type 1/3	177
Proof-of-principle	PLA or PGA + P(CL-LA)	Porous graft reinforced with PLA or PGA mesh	Human (TCPC)	First clinical trials. Graft stenosis identified as primary mode of failure	16–18
	Supramolec-ular polyester	Electrospun microfibrous grafts	Human (TCPC)	Ongoing clinical trial. Feasibility and safety up to 12 months in pediatric patients.	21
	PGS + PCL sheath	Porous electrospun PGS core with PCL reinforcement	Mouse (IA)	Long-term (1 year) functionality in arterial circulation in mice, luminal enlargement	198
	PCL	Electrospun microfibrous grafts	Rat (AA), Pig (CA)	Feasibility of regenerative PCL grafts in arterial circulation vs. ePTFE graft	130, 199
	Hyaluronan-based graft	Coated onto rotating steel core	Rat (AA), Rat (IVC), Pig (CA)	Feasibility of regenerative hyaluronan grafts in arterial and venous circulation	200–202
	In vivo engineered autologous tissue capsule	Fibrocellular matrix created by controlling the FBR to subcutaneously implanted rods	Pig (CA)	Control of FBR to create fibrocellular vascular grafts with sufficient mechanical strength	203, 204
	Decellu-larized SIS	Heparin + VEGF functionalized	Sheep (CA)	Successful in situ regeneration in arterial circulation in large animal	205
		EC/SMC preseeded with fibrin	Sheep (CA)	Successful in situ regeneration in arterial circulation in large animal; cell pre-seeding beneficial but not required	206
	Decellulariz-ed de novo engineered allograft	In vitro tissue-engineered vascular graft from human cells, decellularized	Baboon (AV access)	Grafts maintain functionality in a high flow environment, suitable for hemodialysis.	207
	Decellulariz-ed de novo engineered allograft	In vitro tissue-engineered vascular graft, decellularized	Growing lamb (PA)	Grafts show somatic growth and maintain functionality up to ~1 year follow-up	20
Comorbidities	PCL ± RGD	Nanofibrous grafts	Rat (AA), healthy vs. type II diabetic	Increased complications and impaired regenerative capacity in diabetic vs. healthy rats	184
Graft functiona-lization	UPy-based elastomers	Microfibrous grafts functionalized with UPy-SDF-1α peptide or UPy-PEG + RGD	Rat (AA)	Control over specific cell recruitment and adhesion using supramolecular building blocks	148, 208
	PLCL + heparin/ SP	Microfibrous grafts with covalently bound SP and/or heparin	Rat (subcu-taneous)	Recruitment of MSC and enhanced M2 polarization by heparin/SP functionalization	209, 210

*PGA* polyglycolic acid, *PCL* poly(*ε*-caprolactone), *PLA* poly(*L*-lactic acid), *MCP-*1 monocyte chemoattractant protein-1, *PGS* poly(glycerol sebacate), *SIS* small intestine submucosa, *PLCL* poly (*L*-lactic co-*ε*-caprolactone), *PEO* poly(ethylene oxide), *TPU* thermoplastic polyurethane, *RGD* arginine–glycine–aspartic acid, *UPy* ureido-pyrimidinone, *SP* substance P, *BMC* bone marrow-derived cells, *VEGF* vascular endothelial growth factor, *EC* endothelial cells, *SMC* smooth muscle cells, *FBR* foreign body response, *SDF-1α* stromal cell-derived factor-1α, *PEG* poly(ethylene glycol), *IVC* inferior vena cava, *IA* infrarenal aorta, *AA* abdominal aorta, *CA* carotid artery, *TCPC* total cavo-pulmonary connection, *AV* arteriovenous, *PA* pulmonary artery, *ECM* extracellular matrix, *ePTFE* expanded polytetrafluoroethylene, *MSC* mesenchymal stromal cells

To address these questions, this review provides the state-of-the-art across scientific disciplines regarding the physical, biochemical and biomechanical environmental cues involved in biomaterial-driven in situ tissue regeneration; insights that are essential for the rational design of new, robust immunomodulatory scaffolds for in situ cardiovascular TE. Notwithstanding the promising results obtained with decellularized biological scaffolds, we focus our attention primarily on synthetic-based scaffolds. Synthetic scaffolds have the inherent advantage of being produced under controlled processes, and thus bypassing any reproducibility issues associated with biological scaffolds, as well as being fully tailorable. Therein we take an interdisciplinary point of view, taking lessons from regenerative biology, biomaterials science, immunology, and mechanobiology. To conclude we address the imminent challenges in the field of cardiovascular in situ TE: achieving tissue homeostasis, the development of appropriate preclinical models, and the considerations with respect to stratification for clinical translation.

## Cardiovascular TE—progressing from in vitro to in situ

### Recapitulating the sophisticated biomechanics-dictated cardiovascular microstructures

For both heart valves and blood vessels, the preferred replacement option is a living, adaptive graft. Although the various cardiovascular target tissues (semilunar heart valves, small-diameter arteries, arteriovenous shunts) all come with their own specific requirements, exposure to the hemodynamic environment is the common denominator that poses a major challenge for tissue engineered constructs. Contact with blood implies the need for an endothelium or a surrogate non-thrombogenic layer to prevent thrombus formation, a common complication in synthetic substitutes. Moreover, the hemodynamic loads (e.g., cyclic strains, shear stresses) define the tissue microstructure, leading to a highly-organized and layered structure. For heart valves in particular, it is well-recognized that the valve’s three-layered microstructure (e.g., fibrosa, spongiosa, and ventricularis) is essential to maintain life-long mechanical function.^4, 5^ Many strategies have been followed to engineer such tissues in vitro, as excellently reviewed in more detail elsewhere.^6–8^ However, reproducing the sophisticated native microstructure and its inherent mechanical function has proven to be highly challenging, resulting in varying success. To avoid the complexity of in vitro culture, cardiovascular TE is increasingly progressing towards in situ strategies, relying on the endogenous regenerative capacity of the body. The approach of in situ TE for cardiovascular application is not necessarily new. Already in the 1970’s, reports by Schoen et al. and Sparks describe the exploitation of the foreign body reaction (FBR) to cylindrical implants to create vascular grafts composed of fibrous capsule tissue.^9, 10^ Although the regenerative process in this strategy does not occur directly in situ, these studies demonstrate the potential of FBR-driven endogenous tissue regeneration. By now, this notion has evolved into various strategies to induce endogenous regeneration directly in situ, and the proofs-of-concepts for both vascular and valvular replacements have been achieved, as described in the following.

### In situ tissue engineering of vascular grafts

Among the first reports of vascular in situ tissue engineering is a series of studies by Van der Lei and colleagues, who attempted the use of a biodegradable polyurethane-based scaffold for direct in situ arterial regeneration.^11, 12^ These grafts often developed aneurysms due to lack of mechanical strength of the scaffolds and the authors postulate that pre-seeding with either pre-cultured endothelial cells (EC) or smooth muscle cells (SMC) would be beneficial to accelerate the regenerative process, essentially deviating from the in situ approach as we defined it here. The necessity for pre-seeding of cells into synthetic grafts to stimulate in situ regeneration is still a source of active debate, and the underlying mechanisms remain largely unknown. Pioneering work by the groups of Shin’oka and Breuer using on-the-fly preseeding with bone marrow-derived mononuclear cells (BM-MNC) revealed that vascular neotissue arises from the ingrowth of EC and SMC from the neighboring blood vessel wall and that, contrary to the classic tissue engineering paradigm, preseeded cells do not contribute directly to the vascular neotissue, but instead rapidly disappear after implantation.^13, 14^ Furthermore, vascular neotissue formation was proven to be a host macrophage-mediated regenerative process, with cell seeding being not essential for vascular tissue formation.^15^ Ultimately, the first clinical trials using BM-MNC were shown to improve neotissue formation for replacements of large-bore venous conduits using biodegradable composite polymer scaffolds.^16–18^ However, seeded BM-MNCs were no longer detectable within a few days of implantation, proving that they primarily ameliorated the regenerative process by paracrine signaling to recruit host immune cells, rather than terminally differentiating into functional tissue cells.^19^ Despite the promising clinical outcomes described in these clinical studies, stenosis remained the most prevalent graft-related complication in this low-pressure application.^18^ More recently, Syedain et al. demonstrated proof of somatic growth of an in situ TE pulmonary artery replacement based on decellularized de novo engineered ECM in lambs.^20^ With respect to acellular synthetic grafts, ongoing clinical trials initiated by Xeltis BV demonstrate promising initial results up to 12 months follow-up, using highly porous electrospun grafts from a resorbable supramolecular polyester for total cavo-pulmonary connection in pediatric patients.^21^ Although longer follow-up is warranted, these results are indicative of the clinical potential of in situ TE grafts in the low pressure circulation. Application in the less forgiving high-pressure systemic circulation remains challenging, with aneurysm formation being the most common complication. As underlined in the landmark study by Wu et al., scaffold compliance and appropriate mechanotransduction are particularly important properties for the regeneration of arterial grafts.^22^ Many reports on in situ TE of vascular grafts have contributed to our understanding of the regenerative processes and the influence of the numerous scaffold and host parameters that play a role in this, as summarized in Table 1.

### In situ tissue engineering of heart valves

With respect to heart valves, the most compelling results to date have been achieved using acellular biological scaffolds, such as decellularized porcine small-intestine submucosa,^23, 24^ decellularized allografts,^25–27^ and de novo engineered decellularized ECM.^28, 29^ Decellularized native grafts have been used in various clinical trials and results indicate that these are suitable base materials for in situ TE.^26^ Decellularized de novo engineered valves have been proposed as an alternative to negate the need for a donor valve.^30^ Results by Driessen-Mol et al. demonstrate the feasibility of implanting such valves using minimally invasive delivery techniques leading to extensive recellularization with host cells upon implantation as pulmonary valve replacement in sheep.^29^ However, shortening of leaflets and progressive insufficiency was reported in this study. The Tranquillo group reported the progressive insufficiency of decellularized in vitro engineered valves when implanted as pulmonary valves in a growing lamb model.^31^ In this study, the reported insufficiency was attributed to growth of the valvular root and rather than leaflet shortening. A recent study by the same group demonstrates the sustained functionality of similar valves up to 6 months in the aortic position in sheep.^28^ Interestingly, these valves demonstrated recellularization patterns with vimentin-positive and α-smooth muscle actin (α-SMA)-negative cells, progressing from the root towards the free edge of the valve leaflet, strongly suggesting that these cells originate from the neighboring arterial tissue. Moreover, cellularization mainly occurred on the arterial side of the valve leaflet, while cellularization on the ventricular side was sparse.

To date, experiences of in situ heart valve TE using synthetic-based scaffolds is limited. Weber et al. reported on the implantation of a fast-degrading scaffold based on polyglycolic acid, on-the-fly pre-seeded with BM-MNC, in non-human primates.^32^ However, the rapid degradation of the scaffold did not allow for sufficient neo-tissue formation to maintain functionality. In collaboration with our clinical partners, we demonstrated the proof-of-concept of using a synthetic scaffold for in situ heart valve TE by using a tunable supramolecular elastomer.^33^ These valves demonstrated sustained functionality up to 12 months when implanted in the pulmonary position of sheep, and reported preliminary results demonstrate the compatibility with minimally invasive delivery. In this study, we observed spatiotemporal cellularization patterns similar to the aforementioned results of Syedain et al.,^28^ indicative of gradual regeneration of the valve starting from the hinge region towards the free edge of the leaflet. Prior experience by our group has elucidated that the valve leaflets are acutely colonized with circulatory cells within 1 day of implantation (unpublished data), suggesting that these cells provide the initial cue for neighboring tissue cells to migrate into the valve. Importantly, in our study the degradation of the synthetic scaffold material was observed to be highly localized and was most pronounced in regions with extensive cellularization and neo-tissue formation.^33^ This suggests that the degradation is cell-driven and correlated to ECM formation, which would mean that the structural integrity of the valves is warranted at all times, although further research is needed to validate this. Using derivative technology, Xeltis BV has recently initiated the first clinical trials using an electrospun resorbable supramolecular polymer as pulmonary valve replacement in pediatric patients (XPlore-I and XPlore-II, NCT numbers: NCT02700100, NCT03022708). However, long-term functionality and growth potential of these in situ TE valves remains to be proven, and translation to the clinically most relevant aortic position is all but trivial given the harsh hemodynamic loads in the systemic circulation. Hence, it is important to get a more fundamental understanding of the endogenous regenerative processes and the cues that can be incorporated in a scaffold to modulate these.

## In situ TE - summoning the natural regenerative potential

### Inflammation as the driver of regeneration

The in situ TE approach relies on the regenerative capacity of the body. This natural regenerative capacity is highly species-dependent, and, in adult mammals, scarring is the default repair mechanism in response to trauma rather than functional regeneration. Upon synthetic scaffold implantation, disruption of the tissue structure and subsequent cell damage will initiate an inflammatory response by the host. This is the onset of the classical phased wound healing cascade, culminating in an FBR to the material, as expertly reviewed in detail elsewhere.^34, 35^ Briefly, at the very early stages of implantation, blood-biomaterial interactions lead to the adsorption of endogenous proteins from blood or interstitial fluid to the biomaterial surface. The so formed provisional matrix is rich in mitogens, chemoattractants, cytokines and growth factors which control the subsequent phases of the wound healing and FBR. Following the provisional matrix formation and within the first days after implantation, acute inflammation occurs, with an instantaneous influx of innate immune cells, predominantly neutrophils and monocytes. Chronic inflammation develops as inflammatory stimuli persist at the implant side, with macrophages controlling the microenvironment in cross-talk with lymphocytes, as well as secondary cells, such as fibroblasts, and various stem and progenitor cells, mirroring the cascade of normal wound healing (Fig. 1). Depending on the scaffold properties, the end-stage FBR is characterized either by the accumulation of foreign body giant cells, fibrous encapsulation of the scaffold and eventual graft failure, or by a diminished numbers of macrophages, marked increase in tissue resident cells, and tissue remodeling toward an organized and functional regenerated tissue.

The presence of inflammation can be considered ambivalent; on one hand essential for wound healing, while on the other hand detrimental if any of the phases is disturbed, leading to chronic inflammation, excessive scar tissue formation, and eventual graft failure. Indeed, inflammation is believed to play an all-determining role in biomaterial-driven tissue regeneration. Roh et al. postulated that the in situ regeneration of the inferior vena cava in mice occurred via an inflammation-mediated cascade.^19^ A recent study by our group applying a shielded porous scaffolds in the abdominal aorta of rats revealed a similar phased regenerative cascade, and it was demonstrated that by modulating the initial inflammatory response in the first days after implantation, late-term tissue development was remarkably enhanced 3 months downstream.^36^ Using an immunodeficient mouse model, Hibino et al. proposed that platelet activation and the involvement of Natural Killer cells are critical factors for adverse graft remodeling, leading to vascular graft stenosis.^37^ These results are illustrative of the causality between the initial inflammatory response to an implant and downstream tissue outcome, and show the potential of modulating the long-term regenerative process via the initial scaffold properties.

### The plastic macrophage as a target for immunomodulation

Macrophages have been identified as the commanding cellular mediators in the regenerative process. In several animal studies, macrophage depletion resulted in a significant delay of re-epithelialization, decreased collagen deposition, and impaired healing.^38^ Remarkably, in regenerating species such as salamanders, systemic depletion of macrophages led to failure of growth after limb amputation, while by replenishing the endogenous macrophage population the full regenerative capacity was restored.^39^ Similarly, in injured arteries of rabbits and rats^40^ and in tissue-engineered vascular grafts of mouse models,^15^ systemic macrophage depletion blocked the production of EC, SMC and, ultimately, vascular neotissue.

The functional role of the macrophage is determined by its phenotypic and functional plasticity.^41, 42^ Rather than being purely “big eaters”, inflammatory cells involved in phagocytosis and pathogen clearance, the current consensus is that macrophages are highly plastic cells that adapt their function depending on the tissue microenvironment. As such they can reversely polarize into functionally distinctly different phenotypes, being the so-called pro-inflammatory “M1” phenotype or the pro-wound healing “M2” phenotype. Within the tissue microenvironment, the complex integration of tissue-specific signals, microbial factors, and soluble mediators determines phenotypic changes, and differential activation of these cells. It is now widely accepted that under the crucial influence of soluble mediators secreted by type 1 or type 2 T helper cells (T_H_1 and T_H_2, respectively), macrophages polarize toward the M1 or M2 phenotypes, respectively. More recently a general scheme for macrophage polarization was proposed, based on three different homeostatic activities—host defense, wound healing and immune regulation. It was proposed that M1 and M2a,b,c phenotypes are extremes of a much broader spectrum of functional states^43^ with overlapping M1–M2 characteristics.^44^ Depending on their polarization state, macrophages mediate the formation and remodeling of new tissue by secreting essential growth factors and cytokines that either promote or inhibit functional tissue formation (e.g., transforming growth factor-β, TGF-β; tumor necrosis factor-α, TNF-α; matrix metalloproteinases; platelet-derived growth factor, PDGF).^42, 45^ Besides this regulatory role, it was recently acknowledged that macrophages also directly contribute to tissue formation via producing ECM components themselves, including fibronectin, tropoelastin, various types of collagen and glycosaminoglycans (GAGs) and proteoglycans, such as versican.^46–48^ Moreover, indications for transdifferentiation of macrophages toward the mesenchymal lineage and vice versa can be found in different species and pathologies, but that remains a subject of ample debate.^49–51^ Most important for in situ TE is the notion that the balance of M2/M1 macrophages infiltrating an implanted biomaterial is predictive for long-term tissue outcome.^52^ Consequently, modulation of the macrophage phenotype has become a valuable target to steer the inflammatory response toward functional regeneration.

### Multipotent cells in cross-talk with the inflammatory environment

**Fig. 2:**
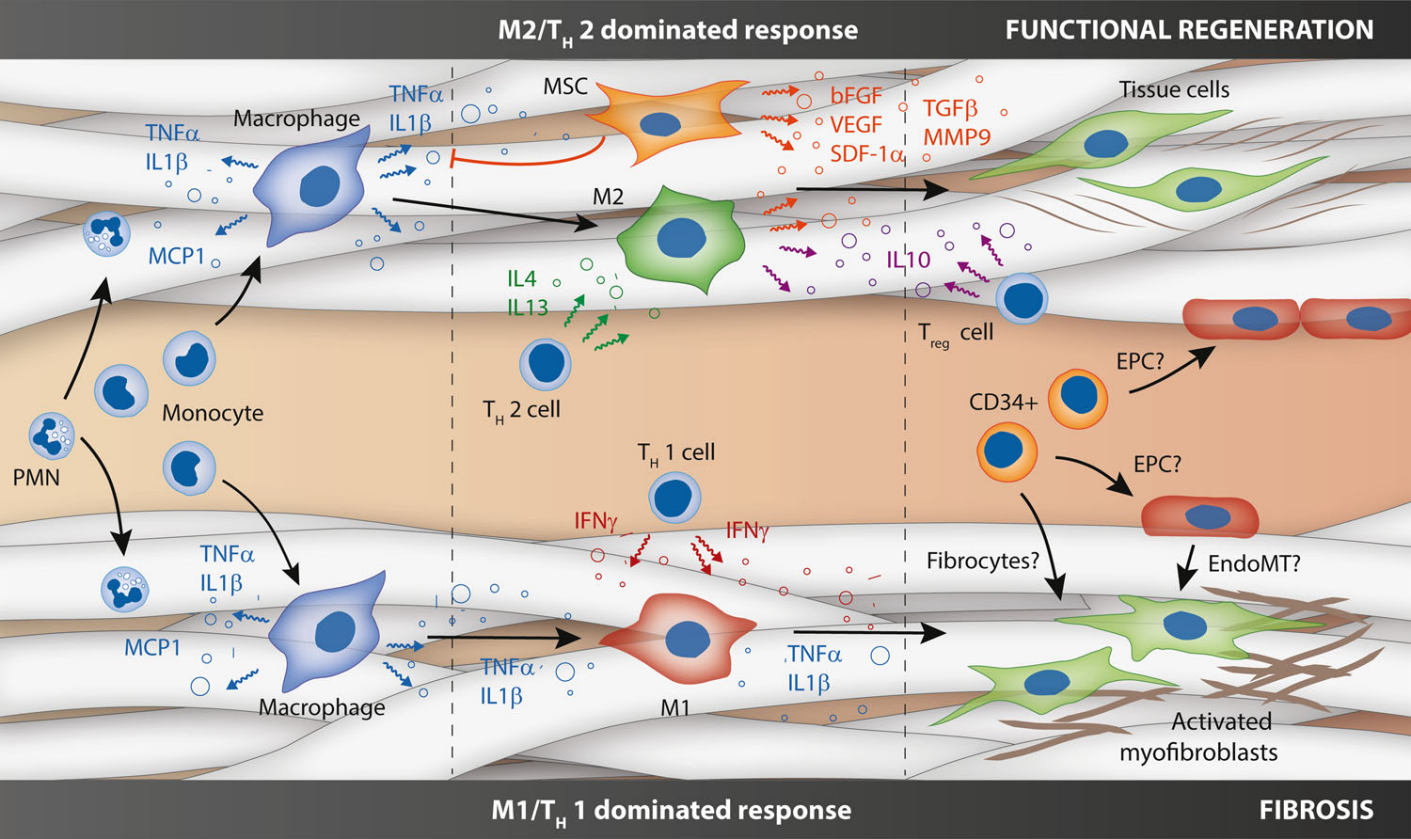
Cartoon of the various (simplified) cell-cell interactions in in situ tissue engineering, as hypothesized based on the state-of-the-art. After the instantaneous response of protein adhesion and platelet activation (not depicted), circulating polymorphonuclear cells and monocytes are recruited to an implanted scaffold in response to various chemokines (e.g., monocyte chemoattractant protein (MCP)-1). Upon activation, the monocytes give rise to macrophages in the scaffold, which are a source of pro-inflammatory factors (e.g., tumor necrosis factor (TNF)-α, Interleukin (IL)‐1β). Depending on the scaffold properties, this is followed by an M1/T_H_ 1 cell dominated response pro-inflammatory response (*bottom*) of an M2/T_H_ 2 cell dominated pro-regenerative response (*top*). The former is characterized by the prolonged presence of M1 macrophages, instigated by T_H_1 cell-secreted pro-inflammatory cytokines, such as interferon-γ. Recruited fibroblasts typically acquire an activated phenotype, producing non-functional cross-linked fibrous scar tissue. In contrast, the pro-regenerative process is dominated by M2 macrophages under influence of T_H_2 cell secreted cytokines (e.g., interleukin (IL)-4 and -13). Mesenchymal stromal cells play an important immunomodulatory role by inhibiting pro-inflammatory factors, such as TNF-α, as well as secreting numerous trophic factors (e.g., basic fibroblast growth factor, bFGF; vascular endothelial growth factor, VEGF; stromal cell-derived factor-1α, SDF-1α; transforming growth factor β, TGF-β; matrix metalloproteinase 9, MMP-9). This biochemical milieu attracts tissue cells and modulates the formation and remodeling of well-organized functional neotissue. Upon scaffold degradation, *T*_reg_ cells inhibit the inflammatory process by secretion of, e.g., IL-10. Homing of circulatory CD34^+^ progenitor cells, such as fibrocytes and endothelial progenitor cells, as well as endothelial-to-mesenchymal transformation may contribute to cellularization and pathophysiological neotissue formation, although these processes are topic of active debate

Supporting the macrophage during the wound healing process, a secondary influx of tissue producing cells, including mature fibroblasts and EC as well as various (circulating) stem/progenitor cells, of both mesenchymal and hematopoietic origin, governs ECM production and remodeling (Fig. 2).^49, 53, 54^ For cardiovascular in situ TE, the origin of mature tissue cells is unknown so far. Although ingrowth of cells from neighboring tissues has been proposed as the predominant route of cellularization in vascular grafts,^14^ its significance for the human situation has been contested.^55^ Given that human cardiovascular tissues have a low natural regenerative potential and a low cellular turnover, regeneration of such tissues is likely to be dependent on the recruitment of cells from high-turnover sites, such as the blood and the bone marrow. Bone marrow-derived progenitors are known to contribute to the valvular interstitial cell population in healthy adult heart valves and homing of progenitors is a normal homeostatic process.^56–58^ The peripheral blood contains various progenitor cell populations, such as endothelial progenitor cells (EPC) and smooth muscle progenitor cells, such as fibrocytes. Although the participation of circulating CD34^+^ progenitors in regeneration is topic of active debate, blood stream-derived cellularization of vascular grafts was proven in dogs, with patchy endothelial coverage with underlying α-SMA^+^ SMCs.^59, 60^ More recently, we demonstrated the regenerative potential of circulatory cells in a rat model of arterial regeneration with a suggested role for CD34^+^ progenitors.^36^ Table 2 summarizes the relevant cell types for in situ TE and their proposed role in the regenerative process.

**Table 2:** Overview of cell types involved in in situ TE and their proposed functions

Cell type		Function	Via
Granulocytes	Neutrophils	Immunomodulation	Release of immunomodulatory factors
	Eosinophils	Biomaterial degradation	Phagocytosis and enzyme/radical excretion
	Basophils		
Monocytes	Classical (CD14 ^+^/ CD16^−^/CCR2 ^+^ )	Immunomodulation	Antigen presentation (e.g., T-cell activation)
	intermediate (CD14 ^+^ /CD16 ^+^ /CCR2^++^)		Production of immunomodulatory cytokines
	non-classical (CD14^dim^/CD16^+^/CCR2^−^)		Differentiation into macrophages or dendritic cells
Macrophages	Host-defense	Immunomodulation	Antigen presentation (e.g. T-cell activation)
	Wound healing	Biomaterial degradation	Immunomodulatory cytokine production
	Immune regulation	Tissue formation and remodeling	Phagocytosis and enzyme/radical secretion
			FBGCs formation
			Production of trophic factors (e.g., TGF-β, VEGF)
			Secretion of ECM components
			Potential transdifferentiation into myofibroblast-like cells
Dendritic cells	Immunogenic	Immunomodulation	Efficient antigen presentation
	Tolerogenic		Immunomodulatory cytokine secretion
Mast cells		Immunomodulation	Production of pro-inflammatory cytokines (e.g., IL-1β, TNF-α, MCP-1)
		Tissue formation and remodeling	Secretion of proteases and anti-inflammatory cytokines (e.g., IL-10, IL-4)
Lymphocytes	T-cells	Immunomodulation	Activation of macrophages to kill antigen bearing cells
	B-cells		Activation of B-cells to produce antibodies
	NK cells		Promoting macrophage-biomaterial adhesion
			Stimulation of FBGC formation
		Tissue formation and remodeling	Influencing macrophage polarization and cytokine production
Mesenchymal stem cells (CD73+/CD90+/CD105+/CD14-/ CD34-/CD45-)		Immunomodulation	Production of mediators to diminish immune cell activation (e.g., IL-10)
		Tissue formation and remodeling	Production of trophic factors (e.g., bFGF, SDF-1α)
			Differentiation into tissue cells
Endothelial progenitor cells (CD34+/VEGFR+/CD133+)	EOCs (myeloid origin)	Tissue formation and remodeling	Production of stimulatory factors for re-endothelialisation and vascularization (e.g., VEGF)
	ECFCs (non-myeloid)		Formation of endothelial cells
			Potential differentiation into myofibroblast-like cell (e.g., EndoMT)
Smooth muscle progenitor cells	CD14^+^/CD105 ^+^ cells	Immunomodulation	Production of immunomodulatory cytokines and growth factors
	Fibrocytes (CD34^+^/CD45^+^/Col I^+^)	Tissue formation and remodeling	Production of ECM proteins
			Secretion of remodeling factors (e.g., MMP’s)

*FBGC* foreign body giant cell, *TGF-β* transforming growth factor, *VEGF* vascular endothelial growth factor, *ECM* extracellular matrix, *IL* interleukin, *TNF-α* tumor necrosis factor alpha, *MCP-1* monocyte chemoattractant protein, *NK cell* natural killer cell, *bFGF* basic fibroblast growth factor, *SDF-1α* stromal cell-derived factor 1 alpha, *EOCs* early-outgrowth cells, *ECFCs* endothelial colony-forming cells, *EndoMT* endothelial-to-mesenchymal transition, *MMP* matrix metalloproteinase, *Col I collagen type I*

Irrespective of cell origin, the interactions between cells in the local scaffold microenvironment are pivotal in the regenerative process (Fig. 2). Local inflammatory reactions contribute to successful regeneration, setting the “soil” for colonizing stem cells, either endogenously recruited or seeded, at the site of injury, as recently reviewed by Forbes and Rosenthal.^61^ In this line, Ballotta et al. described the synergistic expression of trophic factors (e.g., basic fibroblast growth factor, bFGF; stromal cell-derived factor (SDF)-1α) by human mesenchymal stromal cells (MSCs) when activated by peripheral blood mononuclear cells in a 3D electrospun scaffold in hemodynamic conditions.^62^ Moreover, MSCs have been described to exert strong immunomodulatory functions by inhibiting the secretion of inflammatory factors, such as TNF-α and interferon-γ by T_H_1 cells, as well as increasing the secretion of pro-regenerative factors, such as IL-4 and IL-10, by T_H_2 and T_reg_ cells.^63^ Various studies employing in vitro co-cultures have unraveled paracrine signaling mechanisms between macrophages and fibroblasts or SMCs, both in 2D and in 3D scaffolds.^64–68^ Interestingly, Song et al. demonstrated that the macrophage phenotype has an effect on the fibrous matrix production of human fibroblasts via the secretion of either pro-fibrogenic (e.g., TGF-β_1_, PDGF) or anti-fibrogenic and fibrolytic factors (e.g., TNF-α, MMP-7).^69^ Using 3D in vitro co-culture, McBane et al. proposed that monocyte-derived macrophages induce a shift in SMC phenotype form a more synthetic, migratory phenotype to a resting, contractile phenotype.^70^

To conclude, an implanted scaffold serves as an artificial microenvironment that can boost the regenerative capacity of tissue sites with a naturally low regenerative potential, by modulating (1) the local inflammatory environment and (2) the recruitment of stem and progenitor cells to the scene.

## Modulating the regenerative response in the hemodynamic environment

### Rational scaffold design

**Fig. 3:**
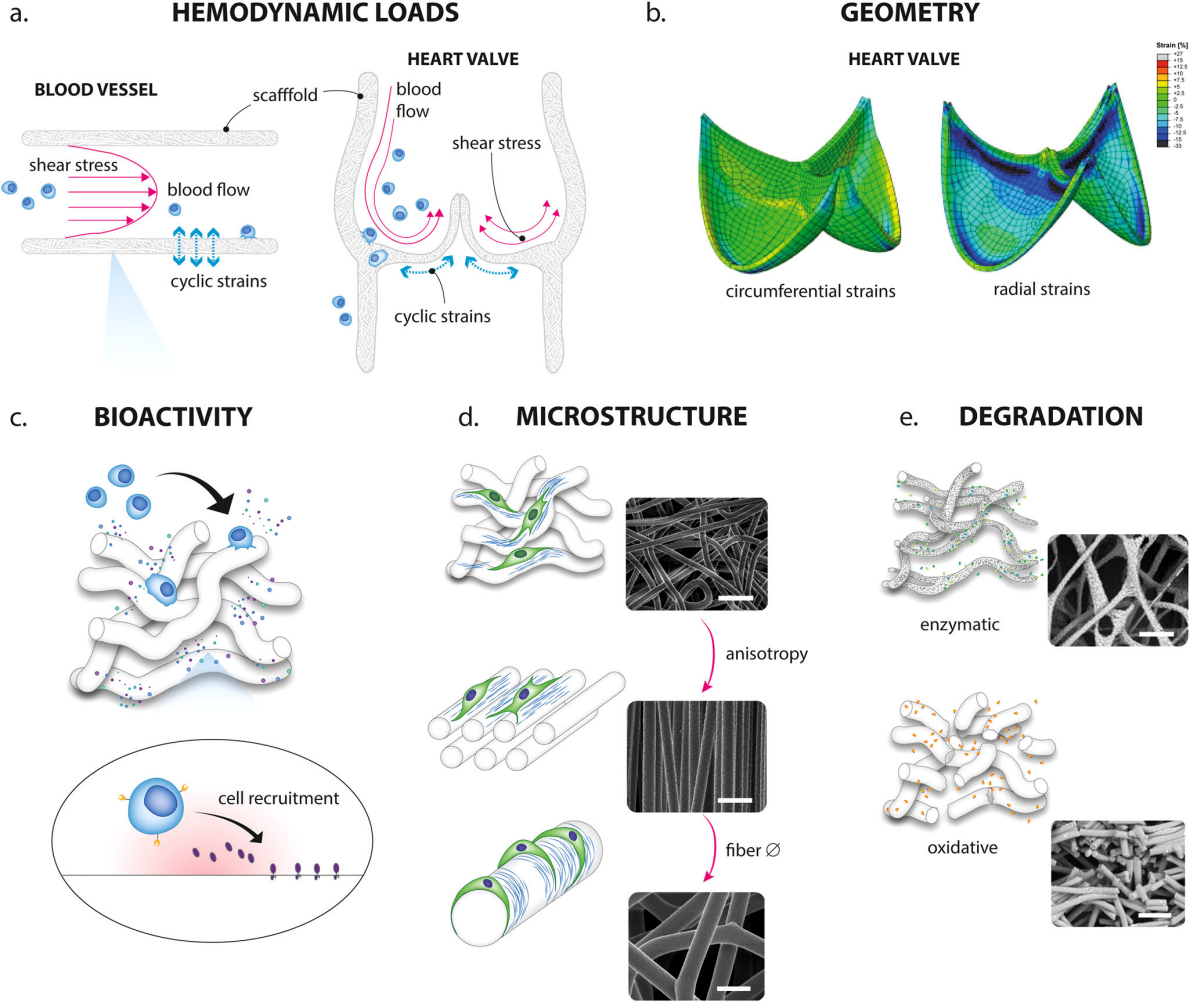
Schematic representation of the design strategies that can be employed to tailor resident cell behavior inside the graft. The transfer of hemodynamic loads (**a**) can be tuned via adaptations in material properties, such as the mechanical properties, geometry (**b**) and microstructure (**d**). Concurrently, cell behavior is defined by interdependent microstructural parameters (e.g., fiber diameter, alignment, pore size and topography; (**d**) and biochemical parameters (e.g., surface chemistry and bio-activation); (**c**). However, local loads and scaffold parameters change in time due to material degradation (**e**) and new tissue formation. Subfigures **b** and **e** are adapted from refs. 107 and 126, respectively

Recent reviews have elaborately described the fundamental potential of modulating the immune response, and more specifically macrophage polarization, using biomaterial properties.^71, 72^ To apply these principles to in situ cardiovascular TE, we should consider the heterogeneity of the infiltrating populations and the interplay between these cells in the mechanically and biochemically dynamic environment. Consequently, in-depth insights are required on the single and combined temporal effects of environmental cues on cell behavior. Rational scaffold design requires interdependent, multi-scale considerations, including micro-mechanical and macromechanical properties, architecture, degradation rate, and bioactivity (Fig. 3).^2^

### Biomechanical stimuli

The biomechanical environment plays an important role in cardiovascular development. Hemodynamic loads dictate the structural composition of valves and vessels, both in physiology and pathology.^73, 74^ For artificial grafts, the continuous exposure to cyclic pressures, shear stresses, and strains require an excellent fatigue behavior of a graft, in particular for heart valves. Moreover, the biomechanical environment dominantly influences the process of cell infiltration and tissue regeneration during in situ TE. To illustrate, implantation of a synthetic graft in the abdominal aorta of mice resulted in a well-organized ECM with native-like elastin content, while a similar graft resulted in only very sparse elastin formation after long-term implantation in the venous circulation.^75, 76^ Transfer of the biomechanical loads (e.g., shear stress, strain) on a cardiovascular scaffold to the infiltrating cells is dependent on the scaffold properties, such as geometry, microstructure, and mechanical properties. Moreover, the local loads change during scaffold degradation and tissue formation. By specifically tailoring these scaffold properties for the aspired clinical application, the hemodynamic load transmission to residing cells can be tuned in order to direct tissue regeneration towards homeostasis (Fig. 3).

#### Shear stress

Shear stresses play an important role in cardiovascular regeneration by affecting cell adhesion, activation and signaling. Moreover, fluid dynamics strongly influence developmental processes, such endothelial-to-mesenchymal transformation,^77, 78^ which are highly relevant for in situ TE strategies, as expertly reviewed elsewhere.^79^ At the cellular level, in vitro studies using 2D fluidic devices have demonstrated shear stress-dependent adhesion of various vascular cells (i.e., SMC, EC, and EPC) to various substrates in controlled shear flow.^80–83^ Shear stresses also have profound effects on the immune response. Increasing shear forces are known to influence platelet activation and adhesion,^84^ as well as the integrin-mediated adhesion and apoptosis of leukocytes.^85, 86^ Correspondingly, in vitro studies in our lab demonstrated that shear stress overruled the effect of biochemical stimulation on specific human monocyte recruitment into a 3D electrospun scaffold loaded with MCP-1.^87^

In vivo, the wall shear stress is notorious determinant of adverse remodeling. Aberrant wall shear stresses at the venous-arteriovenous synthetic graft anastomotic side are known to contribute to neointimal hyperplasia and early graft stenosis.^88^ Interestingly, differential healing of various grafts in dogs was suggested to be implant site-dependent due to variations in fluid dynamics.^89^ Taken together, these results highlight the importance of shear stress for in situ TE. For artificial heart valves, the shear stress profile depends on the valve’s geometrical design,^90, 91^ and as such represents an important design criterion.

#### Cyclic strain

Traditional in vitro TE studies have given us tremendous insights into the causal relationship between strains and ECM formation in 3D scaffolds. Strain is known to be a potent mediator of collagen production and turnover.^92, 93^ Gupta et al. proposed that GAGs and proteoglycan synthesis by valvular cells is dependent on cyclic strain.^94^ Recent studies highlight the importance of physiological strains on the synthesis and organization of the fibrous ECM, and specifically the maturation of elastic fibers.^95, 96^ Importantly, progenitor cells, such as EPCs and MSCs, have been shown to proliferate and differentiate into the cardiovascular cell lineage in response to strain, although the underlying processes of strain-driven differentiation are not well-understood.^97, 98^

For in situ TE, it is important to consider that strains also have an impact on the immunological environment. Straining of monocytes/macrophages was shown to contribute to monocyte-to-macrophage differentiation, a different inflammatory gene expression profile and the selective augmentation of related cytokines, matrix metalloproteinases, and scavenger receptors.^99, 100^ Interestingly, Matheson et al. demonstrated that macrophages display a differential response to uniaxial strain compared to biaxial strain.^101^ Moreover, they showed that cyclic biaxial strain may contribute to biomaterial degradation via augmentation of enzymatic activities (e.g., increased esterase production).^102^ In 3D culture, Ballotta et al. observed that moderate amounts of biaxial strain (7%) may contribute to a more anti-inflammatory macrophage phenotype (M2) when compared to high strains (12%).^103^ In a recent study, Battiston et al. demonstrated that 10% cyclic stretch on a co-culture of monocytes/macrophages and vascular SMC in a polyurethane scaffold contributed to synthesis of collagen type I and III and resulted in improved mechanical tissue properties (elastic modulus, tensile strength).^48^

The fundamental effects of strain on tissue regeneration directly translate to in vivo data of in situ TE vascular grafts. Numerous studies have stressed the importance of graft compliance for successful elastogenesis and tissue regeneration and reorganization.^22, 104, 105^ For electrospun meshes, the macromechanical properties can be predicted by multi-scale modeling, taking into account microstructural elements such as cross-link density, fiber alignment, and fiber diameter.^106^ The resulting strain distribution in the scaffold is determined by the microstructural organization in combination with the macroscopic geometry.^107, 108^ Multi-scale numerical models represent a valuable enabling tool to predict and tune local strains that will be experienced by recruited cells as a result of the hemodynamic loads.

### Scaffold microstructural and material properties

Apart from determining the mechanical behavior, the physical properties of the scaffold have a profound influence on the behavior of colonizing cells. The scaffold microstructure is defined by multiple interdependent parameters, such as the fiber diameter and alignment, pore size, and surface topography and chemistry, and these parameters change during degradation of the scaffold. All these factors are known to have imposing effects on the cellular response to the scaffold, for example, in terms of infiltration, adhesion, and the immunogenic response.

#### Microstructure

The microstructural parameters of fibrous scaffolds provide essential physical cues, such as contact guidance, to infiltrating cells. Using an electrospun scaffold with aligned microfibers, De Jonge et al. demonstrated that newly formed collagen by human myofibroblasts was deposited in the direction of fiber alignment and that the contact-guiding cue overruled the effect of biomechanical stimuli until approximately 80% of the scaffold was resorbed.^109^ This suggests that the formation and organization of neotissue can be directed from the get-go by incorporating anisotropy in the scaffold, such as proposed by Sohier et al.^110^ Interestingly, Fioretta et al. observed a differential effect of electrospun microfibers with varying fiber diameter on cell alignment and ECM deposition between human EPC and mature EC, suggesting that the effect of contact guidance is cell type dependent.^111^

Apart from contact guidance, the microstructure has profound effects on cellular infiltration and the FBR. For electrospun scaffolds, which are most often used in cardiovascular TE, the fiber diameter is linearly correlated to the pore size. As such, fiber diameter and alignment directly influence the cell infiltration depth into the scaffold.^112–114^ Consequently, multi-layered scaffolds have been suggested not only to control the mechanical behavior,^115^ but also to control cell infiltration^116, 117^ (see Table 1). Additionally, it has been shown that electrospun nanofibers minimize blood activation and reduce macrophage activation when compared to microfibers.^118, 119^ Several studies revealed that the mechanism behind which porous materials have improved healing might involve a shift in the polarity of macrophages at the implant site.^120, 121^ Along this line, a positive correlation has been established between M2 polarization and pore size, with an increased expression of angiogenic factors (vascular endothelial growth factor (VEGF), bFGF, TGF-β) with increasing pore size.^122^ Madden et al. proposed a specific “sweet spot” in the scaffold’s microstructural pore size (20–40 μm) to optimally promote favorable M2 polarization.^120^ More specifically, it was demonstrated that this effect is associated with a change in macrophage shape. Upon identifying that M2 macrophages display an elongated, spindle-shaped morphology while M1 macrophages display a round pancake-like shape, McWhorter et al. controlled cell shape and polarization via engineered micro-patterned substrates. Remarkably, shape-induced and cytokine-induced M2 polarization occurred through distinct yet synergizing pathways, suggesting that biochemical cues compliment the effects of geometrical cues.^123^ Other important physical stimuli that may influence macrophage polarization include substrate stiffness, topology and surface chemistry, as recently reviewed in more detail by McWhorter et al.^72^

#### Material degradation

Material degradation will continuously alter the cellular microenvironment, as microstructure and mechanical loading will change in a time-dependent fashion. It is decisive for the success of the scaffold that functional tissue formation and degradation are well-balanced. Prolonged biomaterial presence will result in chronic inflammation and the formation of a tightly cross-linked fibrotic and calcific network that is unable to remodel. Too fast degradation, on the other hand, may result in the loss of structural integrity.

Degradable materials can be degraded within phagosomes after phagocytosis, or eroded via extracellular resorption, with or without the involvement of foreign body giant cells. After degradation, the monomeric components are removed by the natural (metabolic) pathways of the human body.^124^ The biodegradation mechanisms, i.e., (i) hydrolysis, (ii) oxidation, (iii) enzymatic degradation, and (iv) physical degradation are in part dependent on the chemical composition and the morphology of the biomaterial. Hong et al. described the tailored degradation rate of polyurethane scaffolds by partial substitution of polyester segments with polycarbonate segments in the polymer backbone.^125^ Brugmans et al. reported that supramolecular polycaprolactone (PCL)-based polymers are more susceptible to oxidative degradation in comparison to conventional PCL, which is more prone to hydrolytic and enzymatic degradation.^126^

Macrophages have been shown to play a pivotal role in material degradation, via the production of enzymes and reactive oxygen species that can accelerate degradation. Enzymes such as esterases and lipases endogenously produced by macrophages can accelerate the resorption process.^126–128^ Similarly, for the oxidative resorption pathway, macrophages with different polarization states can guide and accelerate the scaffold degradation via secretion of both reactive oxygen species and enzymes.^126, 128^ Understanding of the mechanisms of scaffold degradation is all but trivial, as it determines the extent to which cells (e.g., macrophages) will actively degrade scaffold material. Most importantly, scaffold degradation should be in balance with neotissue formation to maintain mechanical functionality at all times.

Taken together, there are multiple interdependent scaffold parameters, which affect the inflammatory and regenerative response. Table 3 provides an overview of suggested scaffold design principles to achieve an optimal pro-regenerative scaffold biomechanical and physical microenvironments.

**Table 3:** Suggested scaffold design principles regarding physical and biomechanical microenvironment

Parameter	May affect:	Design considerations:
Strain	ECM deposition	Stiffness tailored to:
	Elastogenesis	Obtain appropriate mechanotransduction, which is essential for functional tissue regeneration
	GAGs production	Promote physiological, local and tissue-dependent strains for proliferation and differentiation of tissue producing cells and sustained synthesis and organization of ECM.
	Collagen maturation & organization	Avoid high cyclic strain levels (>8%) as this may induce a proteolytic milieu (pro-inflammatory macrophages polarization)
	Macrophage polarization	
Shear stress	Platelet adhesion and activation	Stiffness and geometry mimicking the native counterpart to:
	Leukocyte adhesion & activation	Promote low shear stresses and adequate cell infiltration
	Cell alignment	Avoid high laminar shear stresses or oscillatory shear stresses, responsible for aberrant cell proliferation, and hyperplasia
	Release kinetics of bioactivated grafts	*Prioritization:* In case of bioactivated grafts, high shear stresses might overrule the release kinetics of the bioactive compounds
Fiber diameter	Cell infiltration	When compared to microfibers, nanofibers:
	ECM deposition	Minimize blood activation
	Encapsulation	Reduce macrophage activation
	Mechanical properties	Reduce fibrous capsule thickness
		Better mimick the nanofibrous structure of ECM
		Present an increased number of fibers per surface unit, and increased strength
Pore size	Encapsulation	Larger pore sizes (20–40 µm) are beneficial over small pore sizes (2–13 μm) for:
	Macrophage polarization	Cell infiltration
	ECM deposition	Expression of angiogenic factors
		Polarization of macrophages into an anti-inflammatory phenotype
		Prevention of calcification
		Improved healing
		*Prioritization:*
		Pore size is a more critical regulator of macrophage polarization compared to the fiber diameter
		Pore-size and cytokine-induced macrophage polarization occurs via distinct yet synergizing pathways. Biophysical cues presented by bio-materials may be used to compliment the effects of geometrical cues
Fiber alignment	Contact guidance to cells	Aligned fibers to be preferred over randomly aligned fibers because of the induced:
	Contact guidance to ECM deposition	Collagen deposition via contact guidance
	Encapsulation	Stimulation of native-like SMC organization via contact guidance
	Mechanical properties	Formation of thinner fibrous capsule
		Layered constructs with different degree of fiber alignments as an effective tool to mimick the hierarchical organization and the variety of local strains exerted in complex native tissues
Topography	Cell adhesion	Rough surfaces to be preferred over smooth surfaces for the reduced adhesion of macrophages, FBGCs accumulation, and fibrotic capsule formation
	FBGCs fomation	
Surface chemistry	Monocyte adhesion	Hydrophilic and anionic surfaces can promote an anti-inflammatory response by:
	Cytokine production	Down regulation of monocyte adhesion
	FBGCs formation	Selective production of anti-inflammatory cytokines
	Encapsulation	Inhibition of IL-4–mediated macrophage fusion into foreign body giant cells (FBGCs)
		*Prioritization:* In terms of fibrous capsule formation, geometric feature (fiber diameter and pore size) are a more critical regulator than topography and surface charge
Material degradation	Macrophage activation	Degradation of the scaffold tailored to:
	Fibrosis	Obtain resorption rates compatible with ECM production
	Calcification	Avoid a too fast degradation, resulting in lack of adequate mechanical support and consequent tissue compaction and retraction avoid too slow degradation, which could promote prolonged macrophage activation, excessive fibrosis and calcification
	Implants degeneration	

*ECM* extracellular matrix, *GAGs* glycosaminoglycans, *SMCs* smooth-muscle cells, *IL-4* interleukin-4, *FBGCs* foreign body giant cells

### Biochemical stimuli

In several animal studies, implantation of vascular grafts in the form of cell-free, bare polymer led to remarkable formation of fully functional neovessel mimicking the native tissue,^22, 129–131^ posing unanswered questions on the necessity for bioactivation for tissue regeneration purposes. Nevertheless, bioactivated scaffolds which house chemotactic and/or trophic factors provide suitable biochemical and physico-chemical cues which, by mimicking the critical aspects of natural healing processes, might accelerate tissue regeneration (Table 4). Also, in patients with cardiovascular diseases, bioactivated scaffolds might augment the limited self-healing capacity by artificially accelerating the proliferation and differentiation of the recruited or implanted cells. On-the-fly preseeding of autologous bone marrow-derived cell fractions into biodegradable synthetic cardiovascular grafts stimulated in situ regeneration of autologous neovessels and valves.^18, 132^ Following studies revealed an interdependent effect on the secretion of trophic factors by preseeded MSC and the inflammatory environment created by circulating immune cells in vitro.^62^ To boost selected signaling molecules or tether endogenously released factors to promote a regenerative microenvironment, biodegradable scaffolds with controlled release of chemoattractants have been developed, which lead to enhanced infiltration of immune cells, de novo tissue formation and reduced fibrosis in several animal models.^19, 36, 133, 134^ Much research has been devoted to the modulation of the inflammatory response via macrophage polarization, with development of several bioactivated materials leading to successful nerve,^135, 136^ bone^137, 138^ and blood vessels regeneration,^139^ both in vitro and in vivo.

**Table 4:** Selection of recent studies employing pre-seeded or biochemically functionalized grafts for regeneration in vitro and in vivo

Goal	Application	In vivo model	Material	Bioactive	Approach	Main findings	Ref.
Crosstalk between preseeded cells and immune cells	Vascular regeneration	Human	PGA + P(CL/LA)	(Cell preseeding: BM-MNC)	Preseeding	No evidence of aneurysm formation, graft rupture, graft infection, or ectopic calcification. Graft stenosis as primary mode of graft failure	18
	Heart valve regeneration	Non-human primates	PGA coated with P4HB	(Cell preseeding: BM-MNC)	Preseeding	Preserved valvular structures and adequate functionality up to 4 weeks post implantation. Substantial cellular remodeling and in-growth resulting in layered, endothelialized tissue	32
	Cardio-vascular regeneration	–	PCL-U4U	(Cell preseeding: BMSC, PBMCs)	Preseeding	PBMC’s secretion of a cocktail of immunomodulatory proteins; MSC’s secretion of trophic factors (MCP-1, VEGF and bFGF); synergistic upregulation of CXCL12 gene expression and bFGF protein production	62
	Vascular regeneration	Mouse	PGA + P(CL/LA)	(Cell preseeding: BMCs)	Preseeding	Early seeded hBMCs depletion; Significant secretion of MCP-1 by seeded cells; MCP-1 guided monocyte infiltration; scaffolds repopulation by SMCs and ECs	19
Phased healing response via temporal release of exogenous growth factors	Vascular regeneration	Rat	PCL	MCP-1	Fibrin coating	At 3 months medial layer with SMCs in an oriented collagen matrix, intimal layer with elastin fibers, and confluent endothelium	36
	Angiogenesis	Mouse (subcutan-eous)	PLGA	SDF1-α	Physical adsorption/ osmotic pumps	SDF1-α guided MSC infiltration in vitro and in vivo; increased angiogenesis and reduced fibrosis in vivo	134
	Heart valve regeneration	Sheep	Bovine pericardium	SDF1-α, fibro-nectin	Coating	Improved homing of primitive cells; native-like matrix deposition; endothelial lining, and complete recellularisation after 5 months	133
	Heart valve regeneration	Dogs	Decellular-ized porcine valves	Fibro-nectin, HGF	Pre-Stirring, pressurization	Accelerated vascularization and endothelialization	211
	Vascular regeneration	Rabbit	Double layered PELCL	VEGF, PDGF	Coaxial electrospinning	Accelerated, sequential proliferation of VEC and VSMC in vitro. Enhanced revascularization and no thrombus formation in vivo	212
	Subcutaneous implantation	Rat	mPEGmA-Ac-TMPTA	Fibro-nectin-derived peptides	Covalent binding	Modulation of inflammatory response, adherent macrophage density, and FBGC formation	213
	Angiogenesis	Mouse	PLG microsphe-res	VEGF, PDGF	Encapsulation, gelification	Stable and sustained improvements in perfusion and angiogenesis	214
	Angiogenesis	Murine	Hydrogels	VEGF	Encapsulation, gelification	Stable and sustained improvements in perfusion and angiogenesis	215
	Angiogenesis	Mouse	GAG hydrogel	VEGF, Ang-1	Non covalent mixing, crosslinking	Stimulation of localized microvessel growth at controlled rates	216
	Angiogenesis	Mouse	Hyaluronan hydrogels	VEGF, KGF	Crosslinking	Stimulation of microvessel growth; appropriate permeability and perfusion; no extravasation of red cells	217
	Angiogenesis	Rat	Dextran hydrogels	EGF, bFGF	Impregnation	Accelerated capillarization and wound healing	218
	Angiogenesis	Rats	Collagen-heparin scaffolds	FGF2, VEGF	Heparin binding	Increased production of collagen type IV and smooth muscle active; early mature vasculature formation	146
Improved/selective cell adhesion	Subcutaneous implantantion	Mouse	PEG based hydrogels	Arg–Gly–Asp (RGD) peptide	Photopolymerization	Modulation of macrophage adhesion via selective engagement of integrins’ bindings	219, 220
	Subcutaneous implantantion	Mouse	Polystyrene microparticles (*in vitro*); PET discs (*in vivo*)	Mac-1, Arg–Gly–Asp (RGD) peptide	Loading in EVA and incubation	Modulation of macrophage adhesion via selective engagement of integrins’ bindings; reduced fibrous capsule thickness	221
	Vascular regeneration	Rat	Supramol-ecular CE-UPy-PCL + UPy-PEG	-	UPy-UPy interactions (quadruple hydrogen bondings)	Reduced cell infiltration in the presence of only 10% of UPy-PEG for cell free vascular grafts.	208
Modulation of inflamma-tory response via macrophage polarization	Vascular regeneration	Mouse	Alginate micro-particles	MCP-1, VEGF	Mixing with collagen/fibronectin gel	Promoted M2 polarization; Increased mature vessel formation via enhanced endothelial and smooth muscle cell recruitment	141
	Nerve regeneration	Rat	Polysulfone/agarose hydrogel scaffolds	IL4	Premixing	Promoted M2 polarization; accelerated downstream axon regeneration	135
	Bone regeneration	Mouse	Decellularized trabecular bone	IFN-γ, IL-4	Physical adsorption (IFNγ), biotin-streptavidin binding (IL4)	Sequential pro-inflammatory M1—anti-inflammatory M2 polarization	138
	Bone regeneration	-	Silk films	IFN-γ, IL-4	Covalent binding	Sequential pro-inflammatory M1 – anti-inflammatory M2 polarization	137
	Vascular regeneration	Rat	Multidomain peptide gels	MCP-1, IL-4	Premixing	MCP-1 dependent cellular infiltration and IL-4 dictated M2 polarization	139
	Intraperitoneal implantation	Mouse	PLG microspheres	IL10	Lentiviral vectors	Cytokine expression modulation of infiltrating leukocytes, decreased inflammation	222
	Nerve regeneration	Rat	Nanofibrous scaffolds	IL-10	Covalent attachment	Promoted M2 polarization	136
	Vascular regeneration	Rat	Supramolecular UPy-PBU	SDF1-α-derived peptide (UPy-modified)	UPy-UPy interactions (quadruple hydrogen bondings)	Reduced expression of inflammatory markers in vitro, promoted macrophages infiltration and early in situ cellularization in vivo	148
	Subcutaneous implantation	Rat	PLCL	Heparin, SP	Covalent attachment	Recruitment of MSC and enhanced M2 polarization by heparin/SP functionalization	209, 210
Tethering of endogenous-ly released factors	Angiogenesis	-	Heparin mimetic peptide nanofibers	VEGF, FGF-2, HGF	Heparin binding	Attachment of heparin-binding cytokines; improved cellular infiltration and angiogenesis	144, 146, 147

*PGA* polyglycolic acid, *PLGA* poly(lactic-co-glycolic acid), *PCL* poly(*ε*-caprolactone), *PCL-U4U* polycaprolactone bisurea, *PLA* poly(L-lactic acid), *PELCL* poly(ethylene glycol)-b-poly(L-lactide-co-*ε*-caprolactone), *P4HB* poly-4-hydroxybutyrate, *UPy* ureido-pyrimidinone, *CE-UPy-PCL* chain-extended UPy-modified poly(*ε*-caprolactone), *PEG* poly(ethylene glycol), *mPEGmA-Ac-TMPTA* mono- methoxy polyethyleneglycol monoacrylate—mPEGmA—acrylic acid—Ac—trimethylolpropane triacrylate—TMPTA-, *PLG* poly(*L*-glutamate), *PLCL* poly (*L*-lactic co-*ε*-caprolactone), *BMC* bone marrow-derived cells, *BM-MNC* bone marrow-derived mononuclear cells, *PBMC* pheripheral blood mononuclear cells, *EC* endothelial cells, *EPCs* endothelial progenitor cells, *SMC* smooth muscle cells, *MCP-1* monocyte chemoattractant protein-1, *RGD* arginine–glycine–aspartic acid, *VEGF* vascular endothelial growth factor, *SDF-1α* stromal cell-derived factor-1α, *IL-4* interleukin-4, *IL-10* interleukin-10, *IFN-γ* interferon- γ, *HGF* hepatocyte growth factor, *PDGF* platelet-derived growth factor, *Ang-1* angiopoietin-1, *KGF* keratinocyte growth factor, *EGF* epidermal growth factor, *bFGF* basic fibroblast growth factor, *FGF-2* fibroblast growth factor-2, *Mac-1* macrophage-1 antigen, *SP* substance P.

Over the last decades various bioactivation methodologies have been developed, with direct adsorption of growth factors to scaffolds and hydrogels representing the simplest approach and the first to be extensively investigated (Table 4).^135, 137, 139–141^ However, when growth factors are administered in their native form, they are susceptible to biodegradation, inactivation due to a very short half-life, and insufficient delivery at the active site. In the search for methods overcoming these disadvantages, encapsulation of growth factors via non-covalent and covalent immobilization were investigated. These delivery systems hold a great deal of promise for localized administration, providing a solubilising and protective environment, minimizing the release to non-target sites, and serving also as an artificial ECM for cell penetration.^130, 140, 142^ Natural occurring GAGs have been identified to bind and modify inflammatory factors, with heparan sulfate being the most well- recognized natural binding site for several cytokines.^35, 143, 144^ In this respect, heparin-conjugated scaffolds^145, 146^ and heparin-mimetic peptide nanofibers^144, 147^ have been proven to indirectly boost cellular infiltration and angiogenesis. Ultimately, recent advances in supramolecular polymers allowed for the development of truly “smart”, cell-responsive scaffolds which can interact with their environment and mediate the host response to the biomaterial, reduce inflammation and promote early in situ re-cellularization.^148^

## Current challenges

**Fig. 4:**
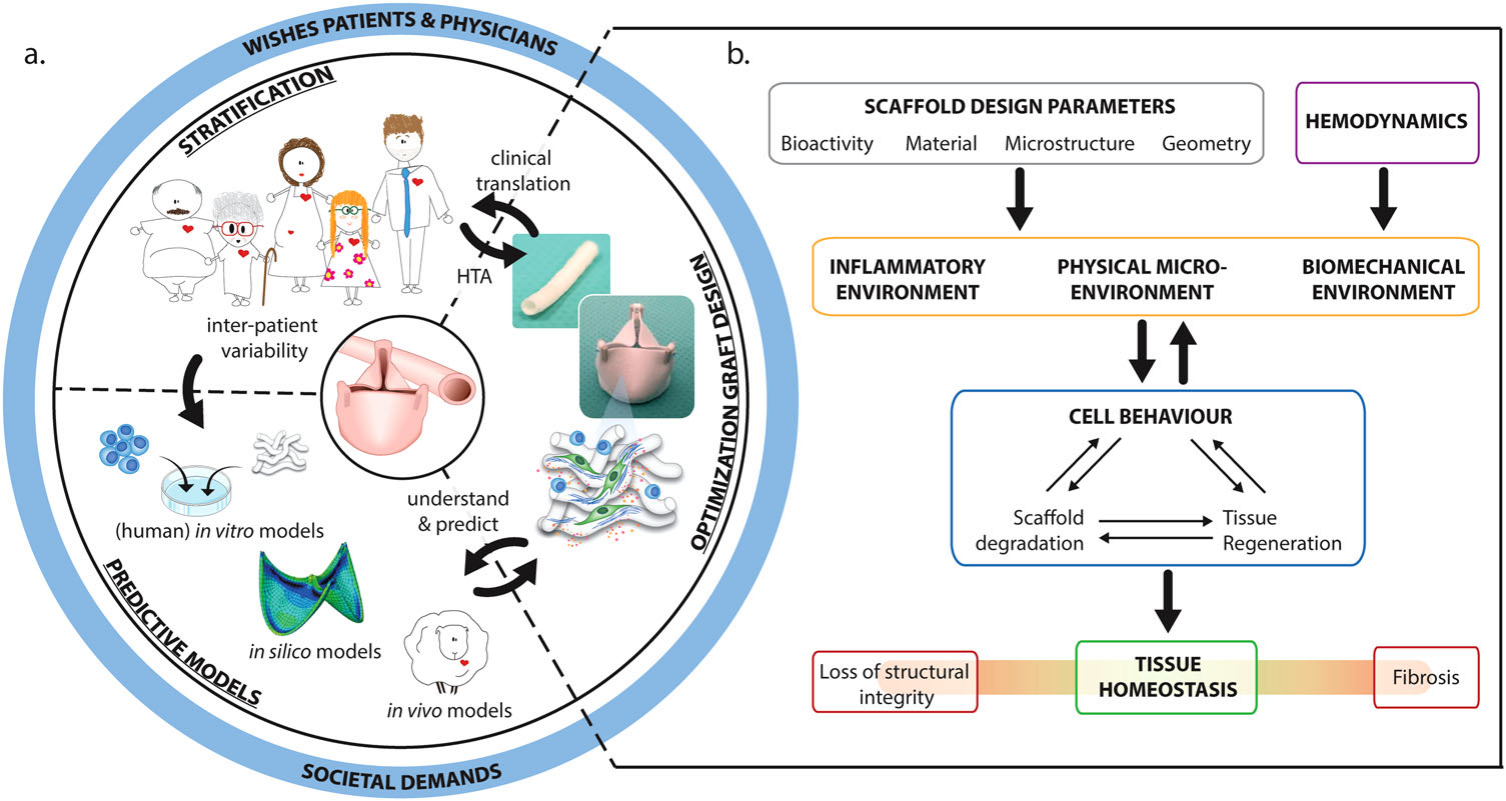
**a** Schematic illustration displaying the three main interdependent challenges faced for successful, robust in-man application of in situ tissue engineered cardiovascular grafts. The development process is represented by a continuous feedback loop between the optimization of the graft design and the development of predictive models to understand and determine long-term in vivo performance, while taking into account graft recipient variability (e.g., age, gender, co-morbidities, and utility). **b** Optimization of graft design is visualized as a flowchart, in which interchangeable scaffold design parameters together with the hemodynamic loads and cells will determine if tissue homeostasis will be reached. Societal demands, including patient and physician wishes, should be taken into consideration during the (early) stages of graft development to determine the added value of these grafts for health care. HTA: Health Technology Assessment

The primary goal for in situ cardiovascular TE is to recapitulate the complex structure and function of the native tissue, in a state of quiescent homeostasis (Fig. 4). Therein, the challenge is not to induce tissue formation, yet the regeneration of functional tissue. This can be achieved by presenting the optimal inflammatory, physical, and biomechanical microenvironments for the colonizing cells. These local microenvironments are constituted by the initial scaffold parameters in the hemodynamic environment, but will change over time due to scaffold degradation and the formation of new tissue. Temporal understanding and control of these processes is one of the main current challenges for in situ TE for cardiovascular applications. Consequently, the development of appropriate models is required in order to predict and tune the regenerative process, and to assess the robustness of the technique depending on patient demographics (Fig. 4).

### Achieving tissue homeostasis

Tissue regeneration is considered the restoration of the original tissue function and structure. In contrast, post-natal wound healing is characterized by abundant deposition of collagen in the form of unorganized bundles within a non-functional matrix, which lacks elastic fibers and functionality: repair, rather than regeneration. The disproportionate accumulation of collagen in cardiovascular tissues, in the form of either a reactive or a reparative fibrosis, further increases stiffness. On the other hand, excessive collagen degradation can tether distortion in tissue architecture, excessive reduction in stiffness, and, ultimately, tissue rupture. For several years, research in the field has focused primarily on collagen deposition, and the possibility to develop cardiovascular grafts of suitable strength, with little regard for elasticity. However, the elastic network represents the hallmark factor distinguishing between the regeneration of functional tissue and fibrous scar tissue. Disturbances in elastin homeostasis have been pinpointed as the underlying causes of valvular grafts failure,^149, 150^ and aneurysma formation.^151^ Although elastin production during cardiovascular in situ TE has been reported, the homeostatic restoration of the native-like, organized elastic network during by adult endogenous cells is a major challenge. This is due to the proteolytic inflammatory milieu, which inhibits elastin expression,^152^ as well as the influence of hemodynamic loads on elastic network formation.^95, 96, 153^ While being essential for the development and remodeling of neotissue, the hemodynamic environment also provides a persistent cue for fibrosis.^154^ Many signaling proteins that are essential to tissue regeneration, such as MCP-1 and TGF-β, are also stimulatory factors for fibrosis. Similarly, on the cellular level, M2 macrophages and fibrocytes, for example, are correlated to both regeneration and fibrosis.^155^ This poses a paradoxal challenge, and the difference between physiological de novo tissue formation and excessive fibrosis is dependent on a rather delicate balance of factors. Developmental principles can provide important lessons for in situ TE. The growth factor profiles in embryonic and post-natal healing are very different,^156, 157^ with embryonic wounds displaying markedly reduced numbers of inflammatory cells compared to adult wounds, due to deficiencies in leukocyte infiltration. Martin et al. proposed that not the macrophage presence itself, but rather the macrophage polarization state influences the balance between scarring and healing, in accordance with previous literature.^158^

In vivo observations emphasize that timely resolution of inflammation is critical. In a recent study, Naito et al. characterized the time course of ECM development in an in situ TE venous graft in mice.^159^ They observed an initial surge in the production of fibrillary collagen, which was postulated to be part of an initial cellular FBR to isolate the polymer. This response was alleviated upon degradation of the scaffold, after which other ECM proteins, such as GAGs, elastin, and collagen type IV increased proportionally. As a result, the mechanical properties of the graft converged to those of the native vein.^160^ This was attributed to the transient changes in mechanotransduction of hemodynamic loads to the cells. In correspondence, delayed degradation and lack of mechanotransduction was pinpointed as the cause of insufficient tissue regeneration in arterial grafts in mice.^161^ Similarly, appropriate mechanotransduction and timely degradation of the scaffold were hypothesized to be the key factors underlying the successful in situ regeneration of a neoartery in rats.^22^ Accordingly, long-term follow-up of arterial PCL grafts in rats revealed extensive chondroid metaplasia, which was likely caused by the persistent presence of the PCL scaffold and poor mechanotransduction, up to 18-month follow-up.^131^ Sugiyura recently demonstrated that calcification of arterial grafts in mice could be avoided in fast-degrading grafts, in contrast to slow-degrading grafts.^162^ Correspondingly, Wu et al. advocated the importance of rapid resorption and appropriate mechanotransduction for the remodeling of fast degrading synthetic grafts toward physiological-like neoarteries.^22^

In summary, all these results corroborate that degradation is inextricably correlated to tissue formation and remodeling. The timely resolution of inflammation is a pivotal factor in the process of de novo tissue formation and the prevention of adverse remodeling. This resolution is governed by the biochemical and biomechanical microenvironment, which may be concerted by well-timed degradation of the synthetic scaffold (see also Table 3).

### Development of predictive models

Perhaps one of the most underexposed challenges for in situ cardiovascular TE is the development of appropriate models to predict the long-term in vivo performance of grafts. When employed appropriately, animal models are a vital source of information to study and mimic the complex regenerative processes in vivo. Small animals (mice, rats) in particular are being used extensively to answer preliminary research questions in the developmental phase of new prostheses (e.g., regarding biocompatibility, material choice, and device design). However, although the bulk of animal studies for in situ vascular regeneration reports on promising patency and endothelialization rates, both the underlying mechanisms and the timescales are subject to strong interspecies variations, as well as implant-site variations,^89, 163^ which may mislead their conclusions in terms of clinical application. It is often overlooked that the predictive value of an animal model is only as useful as the context in which it is interpreted.^164^ For example, the mechanisms governing the cellularization of the graft with functional tissue cells (i.e., EC and SMC) differ between species. Endothelialization of vascular grafts in mice and rats is characterized by a rapid, progressive transanastomic overgrowth,^14^ which is typically not observed in humans, even after prolonged implantation periods. Therefore, refined animal models have recently been proposed by ourselves^36^ and others.^165^ Differences in critical hemodynamic parameters (e.g., shear stress) between different animal models and humans may further decrease the value and predictability of animal models.^166^

Another important consideration in the use of animal models is the immunological variance between species, especially considering that the process of in situ TE is highly dependent on the immune response to the implanted biomaterial. It has been demonstrated that the genomic response to acute inflammatory stimuli in mice poorly correlates to the human conditions, as well as between different mouse models.^167^ Genomic comparison of mice and human monocyte subpopulations has revealed that, although general expression patterns are conserved, significant and even opposing functional differences exist between species.^168^ Similarly, the characterization of the subsets based on marker expression is species-dependent. This calls for species-specific immunological marker panels to study the function of specific leukocyte populations in vivo.

Novel in vitro and in silico models that aim to predict the biomaterial-dependent host response are starting to gain attention as of recent.^113, 169–171^ Various recent studies defined the macrophage response and the resulting cytokine/chemokine profile (e.g., IL-6, TGF-β, TNF-α, MMP-9 secretion) as predictive parameters for the long-term host response on a biomaterial.^170, 172, 173^ In a recent study, Wolf et al. evaluated the static in vitro response of human peripheral blood mononuclear cells toward various synthetic and biological materials in vitro in terms of macrophage M1/M2 ratio and secretion of a small set of signaling proteins (MMP-2, MMP-9, IL-6, and interferon-γ-induced protein (IP)-10). Coupled with in silico principle component analysis, the in vitro data could be successfully correlated to the long-term tissue outcomes of the same materials implanted subcutaneously in a animal model.^170^ Enayati et al. highlighted the potential of a fibroblast-macrophage coculture model to increase the predictive value of in vitro models.^174^ Adding physiological complexity, bioengineered vascular tissues may serve as suitable in vitro models, as recently reviewed by Wolf et al.^175^

Taken together, advanced in vitro and in silico models mimicking the principal components of the host response to an implanted biomaterial or even “humanized” animal models in synergy with systematic in vitro studies, might represent an excellent and potentially superior alternative to animal models.

### Stratification for clinical translation

Apart from aforementioned inter-species differences, patient-to-patient variability poses a challenge for stratification of in situ TE therapies. Even without considering any graft parameters, in situ TE is completely dependent on the natural regenerative potential of the graft recipient. The wound healing response, and thereby the intrinsic regenerative capacity are highly variable between patients, and even between healthy individuals. Mammalian wound healing is prone to genetic variability.^176^ Khosravi et al. who recently reported significant functional diversity in the long-term remodeling of identical arterial grafts, even in healthy laboratory animals.^177^ There is a strong natural variability in both the innate and adaptive immune response among humans, which, to a large extent, can be attributed to differences in age or gender.^178–181^ Consequently, young people and pre-menopausal women are known to have an increased risk of scarring than men and elderly, which is attributable to variations in the immune system.^156^ Common comorbidities of cardiovascular patients include for example diabetes and chronic kidney failure, further contributing to the variability of the regenerative capacity and thereby, the applicability of such techniques for specific patient cohorts. Krawiec et al. reported an increased risk of stenosis in vascular grafts engineered from human cells from diabetic patients, which was suggested to be due to a reduced remodeling capacity.^182, 183^ Wang et al. reported a significantly reduced regenerative capacity of diabetic rats.^184^ Together, these findings highlight the importance of risk stratification and the potential need for a personalized approach in translating these therapies to the clinic. Moreover, these are important considerations when selecting the appropriate patient cohort for first-in-man studies. Given the limitations of current alternative treatments, in situ TE would be most beneficial for children and young adults. Hence, pulmonary valved conduits for children with complex congenital heart disease would represent a most valuable first-in-man target, as is the case for the currently ongoing clinical trials by Xeltis BV.

Moreover, to translate toward in-man clinical application, it is important to remain focused on the clinical requirements, the wishes and needs of patients and physicians (cardiothoracic surgeons) as well as the societal demands. Given the societal demand for better, sustainable and more efficient health care it is important to search for strategies that have a higher return of investment and a reduced time to the market. Early Health Technology Assessment (HTA) is emerging as a research field focusing on the evaluation of medical, economic, social and ethical implications of a new medical device to determine the added value for health care.^185, 186^ As an example, conceptual models can be developed to determine the graft requirements to become cost-effective compared to golden standard treatment methods.^187^ Hence, these assessments can be used to continuously prioritize and guide design choices during development, to optimally introduce and use the grafts in the clinic. For example, patients with a limited natural regenerative capacity might benefit from the local incorporation of bioactive factors into an in situ TE scaffold (Table 4), and/or complementary systemic therapies, such as drugs to initiate stem cell mobilization.^188^

## Conclusion

In situ TE of blood vessels and heart valves using resorbable synthetic grafts is rapidly progressing and first clinical trials are exemplary of its clinical potential. Here we described the underlying principles of biomaterial-driven regeneration, initiated by the host response to the material and governed by the interplay of immune cells (e.g., macrophages), stem/progenitor cells and tissue cells in the scaffold microenvironment. In enhancing our multi-disciplinary understanding of the fundamental processes underlying successful endogenous regeneration, it is important to consider the interdependent role of physical and biochemical cues in this process, hinging around the biomechanical cues exerted by the hemodynamic environment. The development of advanced predictive models will contribute to stratification of in situ cardiovascular TE as a robust clinical therapy.

### Data availability

Data sharing not applicable to this article as no data sets were generated or analyzed during the current study.
